# Homocysteine Induces Apoptosis of Rat Hippocampal Neurons by Inhibiting 14-3-3ε Expression and Activating Calcineurin

**DOI:** 10.1371/journal.pone.0048247

**Published:** 2012-11-06

**Authors:** Jing Wang, Xue Bai, Yongxing Chen, Yanxin Zhao, Xueyuan Liu

**Affiliations:** Department of Neurology, Shanghai Tongji University Affiliated Tenth People’s Hospital, Shanghai, China; University of Iowa, United States of America

## Abstract

A high level of plasma homocysteine (Hcy) increases the risk for neurodegenerative diseases. One such disorder is Alzheimer’s disease, which involves marked neuronal apoptosis of unknown etiology. This study shows that Hcy inhibits expression of 14-3-3ε and activates calcineurin in rat hippocampal neurons in a dose-dependent manner. Calcineurin-mediated Bad dephosphorylation, which is blocked by calcineurin inhibition and Ca^2+^ chelation, causes mitochondrial translocation of Bad and apoptosis; this step in the apoptotic pathway is synergistically blocked by calcineurin inhibition and overexpression of 14-3-3ε. These findings demonstrated that calcineurin activation and downregulation of 14-3-3ε may be one of the mechanisms of Hcy-induced apoptosis of hippocampal neurons.

## Introduction

Homocysteine (Hcy) is a non-protein amino acid, or thiol-containing amino, which is derived from methionine [1]. Hcy can be remethylated to methionine by folic acid-dependent enzymes and can be catabolized to form cysteine by cystathionine-β-synthetase, a vitamin B6-dependent enzyme.

Elevated plasma Hcy levels have been associated with high incidence of atherosclerotic [2] and neurodegenerative disorders, such as Alzheimer’s disease and dementia [3], [4], [5], [6], [7]. Evidence shows that Hcy is toxic to neuronal cells both *in vitro* and *in vivo* [8], [9] and can cause calcium influx, and neuronal apoptosis [10]. While inducing apoptosis is an important effect of Hcy in neuronal cells [8], [11], the mechanism involved is, however, not yet well understood.

**Figure 1:**
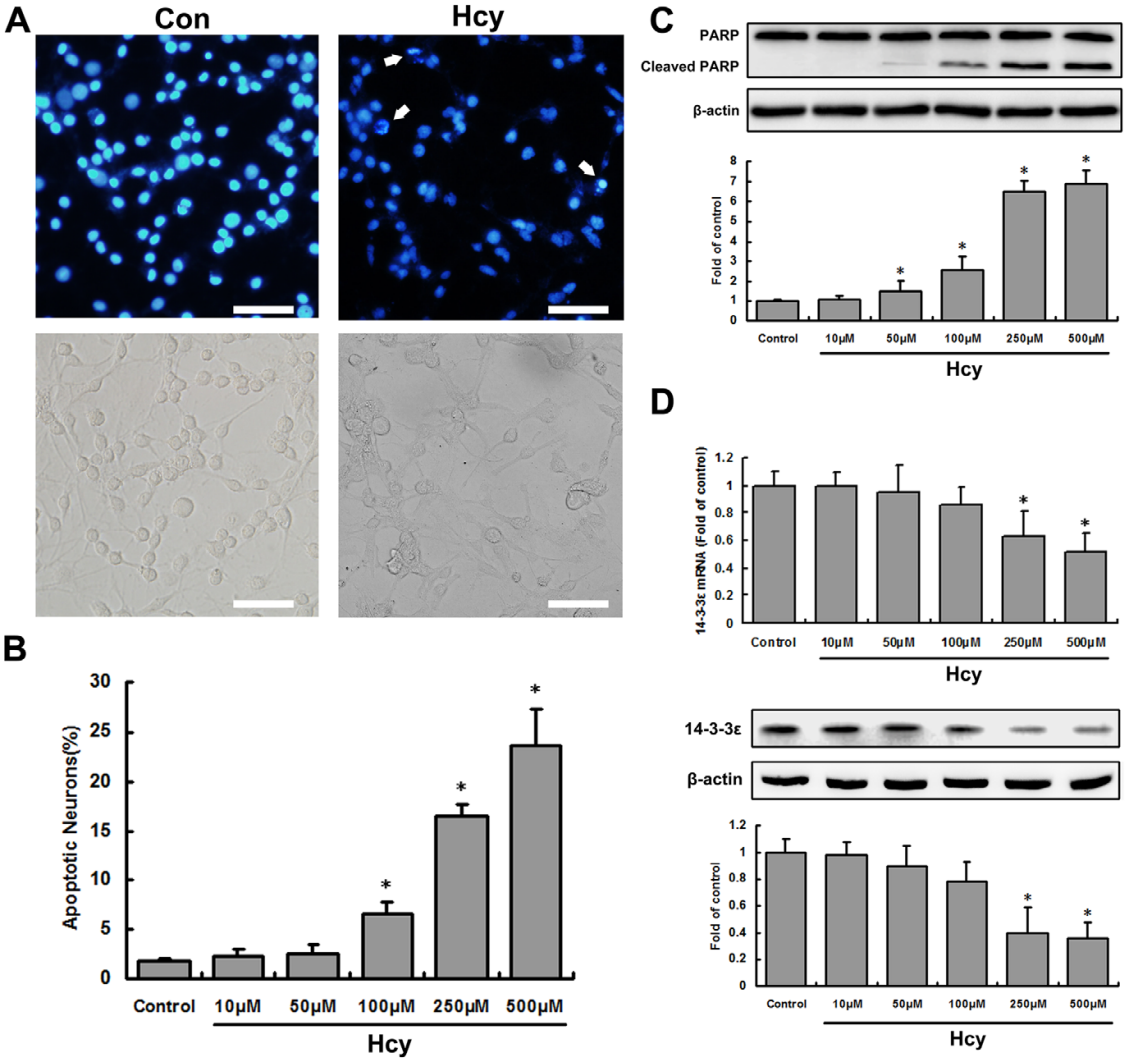
Hcy induces apoptosis and suppresses 14-3-3ε expression in cultured hippocampal neurons. A, Cultures were exposed for 24 h to either saline (*Con*) or 500 µM Hcy (*Hcy*) and were subsequently stained with the fluorescent DNA-binding dye Hoechst 33342 (*top*) or photographed under phase-contrast optics (*bottom*). Note the nuclear DNA condensation and fragmentation in neurons exposed to Hcy (arrow). Scale bar: 100 µm. B, Cultures were exposed to the indicated concentrations of Hcy for 24 h, and the percentages of neurons with apoptotic nuclei were quantified. Values are the mean ±S.E.M. of counts made in four to six cultures. C, Cells were treated as in B, and the levels of cleaved PARP were determined by western blotting. Top, representative western blots. Bottom, densitometric analysis of cleaved PARP normalized by β-actin (*n* = 3). D, 14-3-3ε mRNA and protein was measured by real-time quantitative PCR (*top*) and western blotting (*mid and bottom*), respectively. *P<0.05 vs. control.

In an effort to identify factors contributing to the neurotoxicity of Hcy, we previously studied protein alterations in rat hippocampal neurons by the quantitative mass-tag labeling proteomic technique iTRAQ and mass spectrometry. We found that the levels of 14-3-3ε, a member of the 14-3-3 family, were significantly reduced after treatment with Hcy.

The 14-3-3 family have seven 14-3-3 isoforms in mammals, which together comprise 1% of total brain protein, and these proteins participate in many cellular functions by binding to specific phosphorylated sites on diverse target proteins [12], [13]. 14-3-3 is involved in the regulation of apoptosis and 14-3-3 depletion can lead to activation of pro-apoptotic factors [14]. Increased 14-3-3 expression has been proven to show a strong neuroprotective effect in multiple cellular and animal models of the neurodegenerative disorder Parkinson’s disease [15]. 14-3-3 suppress apoptosis mainly through 14-3-3-mediated sequestration of Bad, a pro-apoptotic client protein that has been linked to mitochondrial apoptosis [12], [16]. However whether 14-3-3s play a role or are connected to Ca^2+^ influx in apoptosis induced by Hcy is unknown.

Previous studies have shown that over-stimulation of *N*-methyl-D-aspartate (NMDA) receptors, oxidative stress and cytochrome *c* release are involved in apoptosis induced by Hcy [8], [9], [17], [18], [19]. Over-stimulation of NMDA receptors leads to increases in the levels of cytoplasmic Ca^2+^, and Ca^2+^ influx induced by L-glutamate can cause mitochondrial apoptosis through calcineurin dephosphorylation of Bad in hippocampal neurons [20]. However, studies linking calcineurin-mediated Bad dephosphorylation to apoptosis in Hcy-treated neural cells are lacking.

As it appears that Bad-mediated mitochondrial apoptosis is involved in the neurotoxicity of Hcy, the purpose of the present study was to study the effects of 14-3-3ε and to determine whether Ca^2+^-induced dephosphorylation of Bad was also involved in the Hcy-induced apoptosis.

## Materials and Methods

### Hippocampal Cell Cultures and Experimental Treatments

Rat primary hippocampal neuron cultures were prepared as described previously [21] with slight modifications. Briefly, the brains were removed from embryonic day 18 Sprague–Dawley rat embryos. The hippocampi were dissected and mechanically disaggregated by gentle trituration using a Pasteur pipette. Dissociated cells were seeded at a density of 1×10^6^ cells/cm^2^ on sterile poly-L-lysine-coated (15 mg/ml) 12 mm plates and maintained in growth medium at 37°C with 5% CO_2_. The growth medium consisted of Neurobasal/B27 (Invitrogen, Carlsbad, CA) supplemented with 0.5 mM glutamine and 25 µM glutamate. All experiments were performed in 8–10-day-old cultures. L-Homocysteine (Sigma, St. Louis, MO), cyclosporin A (CsA; Sigma) and BAPTA-AM [1,2-Bis(2-aminophenoxy)ethane-N,N,N′,N′-tetraacetic acid tetrakis(acetoxymethyl ester), Sigma] were prepared as concentrated stocks in sterile water, pH 7.2, and experimental treatments were added to cultures by dilution in culture medium. All animal experiments were approved by the Animal Research Committee at Shanghai Tongji University and were carried out in accordance with established International Guiding Principles for Animal Research.

**Figure 2:**
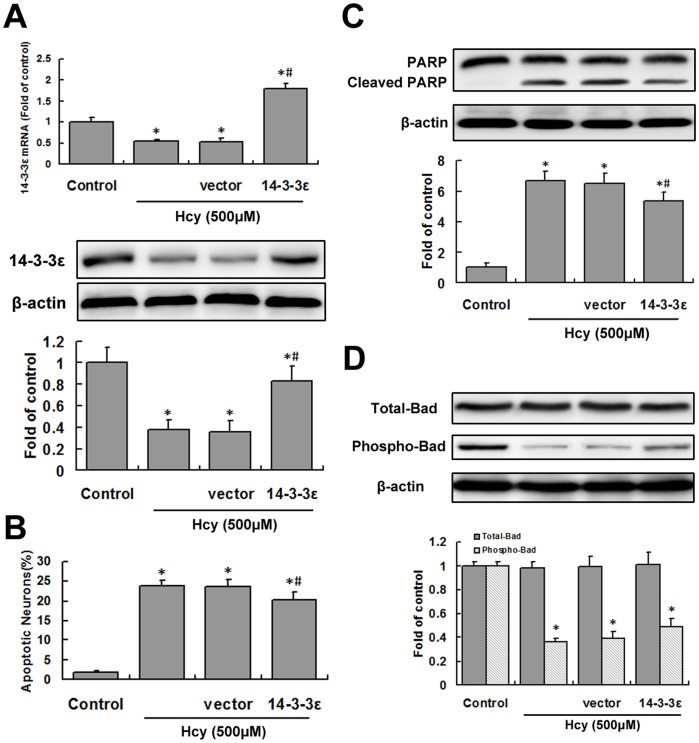
The effect of lentiviral 14-3-3ε transduction on apoptosis in hippocampal neurons. Hippocampal neurons transduced with lentiviral 14-3-3ε for 48 h were treated with Hcy for 24 h. A, 14-3-3ε mRNA and proteins were analyzed by real-time quantitative PCR or western blotting, respectively. B, The percentages of neurons with apoptotic nuclei (Hoechst staining) were quantified. C, Cleaved PARP was determined by western blotting. D, Total Bad and phospho-Bad levels were assessed by western blotting and densitometric analysis. *P<0.05 vs. control. ^#^ *P*<0.05 vs. Hcy treatment group.

### Assessment of Apoptosis

To quantify apoptosis, cells were fixed in 4% paraformaldehyde and stained with the fluorescent DNA-binding dye Hoechst 33258 following the manufacturer’s protocol (Apoptosis Hoechst staining kit, Beyotime Biotechnology, Jiangsu, China). Hoechst-stained cells were visualized and photographed under a fluorescence microscope (Olympus; Shinjuku-ku, Tokyo, Japan) at an excitation wavelength of 330–380 nm. Three hundred cells/culture were counted, and counts were performed in at least four separate cultures/treatment condition. Those cells with condensed and fragmented nuclear chromatin were considered apoptotic and the percentage of apoptotic cells in each culture was determined.

### Real-time Quantitative Polymerase Chain Reaction

For real-time quantitative polymerase chain reaction (PCR) analysis of 14-3-3ε mRNA levels, an RNeasy Mini Kit (Qiagen) was used to extract RNA from cells. Contaminating DNA was removed by DNase I digestion. cDNA was generated from 1 µg total RNA, and 1/20^th^ of the cDNA mixture was used for quantitative reverse transcription in an iCycler (Bio-Rad, Munich, Germany). A typical 25 µl reaction mixture contained 12.5 µl 2 ×SYBR Green QPCR Master Mix (Applied Biosystems, Foster City, CA), 11 µl water, 0.5 µl template and 400 nM primers (0.5 µl each from stocks): 14-3-3ε, 5′-AAGATGATTCGGGAGTACCGGCAA-3′(forward) and 5′-TCCTGTGGCAAACTCAGCCAGATA-3′ (reverse); β-actin, 5′-TTGCTGACAGGATGCAGAAGGAGA-3′(forward) and.

5′-ACTCCTGCTTGCTGATCCACATCT-3′ (reverse). Quantitation of mRNA expression was carried out on ABI Prism 7900 Sequence Detection system (Applied Biosystems, Foster City, CA) using the following conditions: 10 min at 95°C (hold), 15 s at 95°C, and 1 min at 60°C (45 cycles). All reactions were carried out in triplicate to reduce variation. Data normalization was accomplished using the endogenous control (β-actin), and the normalized values were subjected to a 2^−△△*Ct*^ formula to calculate the fold change between the control and experiment groups.

**Figure 3:**
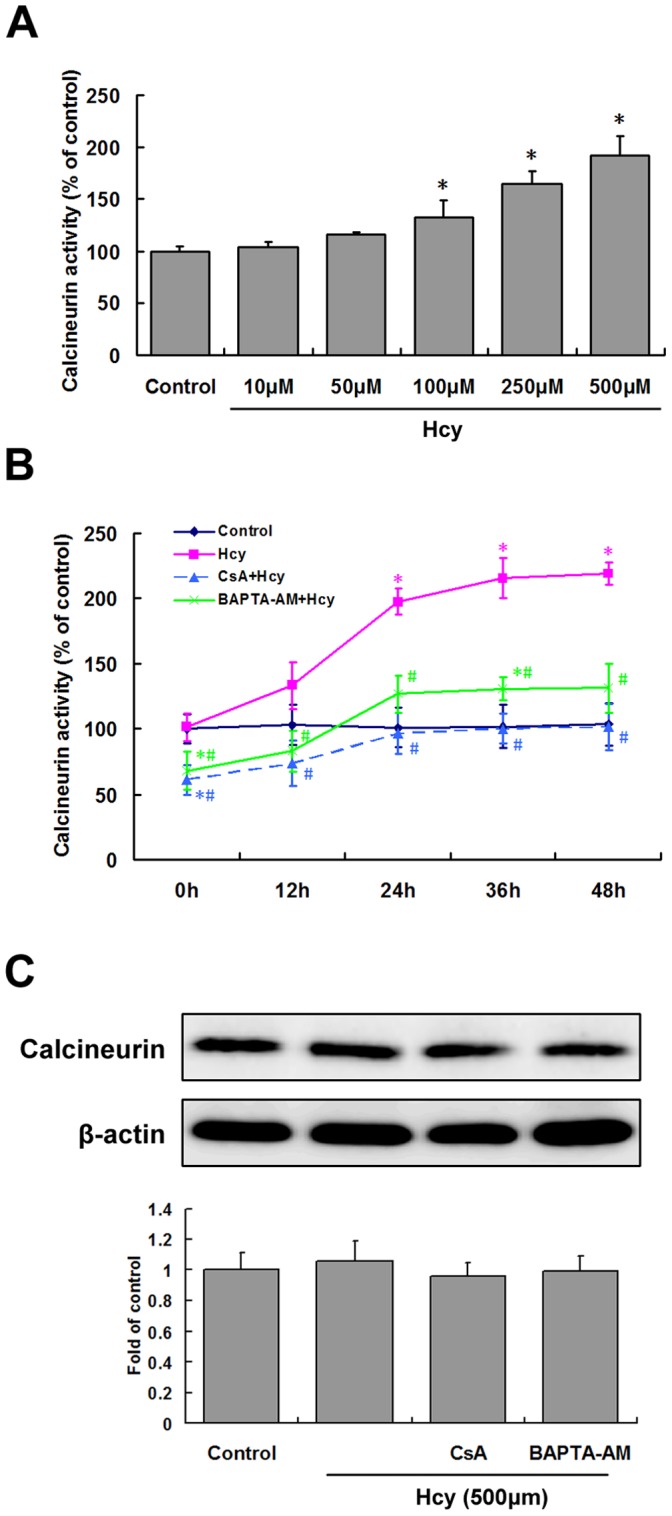
Activation of calcineurin by Hcy and inhibition by specific blockers. A, Cultures were exposed to the indicated concentrations of Hcy (*Hcy*) for 24 h, and cellular calcineurin activity was then determined. B, Cells were pretreated with 1 µM CsA or 10 µM BAPTA-AM for 30 min before exposed to 500 µM Hcy (*Hcy*). Cellular calcineurin activity was measured at the indicated time periods after the addition of Hcy to the cells. Values represents the mean ± S.E.M. of four or more assays. C, Cells were pretreated with 1 µM CsA or 10 µM BAPTA-AM for 30 min before exposed to 500 µM Hcy (*Hcy*) for 24h. Calcineurin A (Calcineurin) protein level was determined by western blotting. Top, representative western blots. Bottom, densitometric analysis. *P<0.05 vs. control. ^#^ *P*<0.05 vs. Hcy treatment group.

### Western Blots

We used western blotting to examine changes in Bad in subcellular fractions where it is reported to be localized and/or translocated. Protein from enriched fractions of mitochondria and cytosol was extracted as described previously [22]. Briefly, after different treatments, hippocampal neurons were harvested in TBS (Tris-buffered saline) and centrifuged at 500 *g*. Cell pellets were resuspended in isotonic mitochondrial buffer (MB; 210 mM mannitol/70 mM sucrose/1 mM EDTA/10 mM HEPES, pH 7.5), supplemented with a protease inhibitor (Roche, Indianapolis, IN) and homogenized with 30 strokes with a Dounce homogenizer. Samples were centrifuged at 500 *g* for 5 min at 4°C and the resulting supernatant was centrifuged at 10,000 *g* for 30 min at 4°C. The heavy membrane pellet represented the mitochondrial fraction while the supernatants contained the cytosolic fraction. Total hippocampal neuronal protein was extracted by centrifugation at 13,000 *g* for 5 min following lysing in the extraction buffer (50 mM Tris, pH 7.4, 150 mM NaCl, 1 mM EDTA, 1% Triton X-100, 0.5% sodium deoxycholate, 30 mM NaF, 1 mM protease inhibitor).

Equal quantities of protein were separated by sodium dodecyl sulfate-polyacrylamide gel electrophoresis (SDS-PAGE), followed by transfer to nitrocellulose membranes. The membrane was preincubated in blocking buffer [TBS containing 5% (w/v) non-fat dried milk] for 1 h at room temperature (26°C) and then probed with a primary antibody overnight at 4°C. After washing with buffer, the blots were incubated with the secondary antibodies (peroxidase-conjugated anti mouse or anti-rabbit IgG) at room temperature for 1 h. Blots were developed using the enhanced chemiluminescence system (Amersham, Arlington Heights, IL), following the manufacturer’s instructions. Densitometric analysis was performed using ImageJ software. Anti-rat primary antibodies used were as follows: 14-3-3ε polyclonal antibody, β-actin monoclonal antibody, and cytochrome *c* oxidase subunit 4 antibody (all from Santa Cruz Biotechnology, Santa Cruz, CA, U.S.A.), and PARP polyclonal antibody (Cell Signaling Technology, Inc. Danvers, MA, USA), and calcineurin A monoclonal antibody, Bad monoclonal antibody, and anti-rat Phospho-Bad polyclonal antibody (all from Abcam plc. Cambridge, MA, USA).

**Figure 4:**
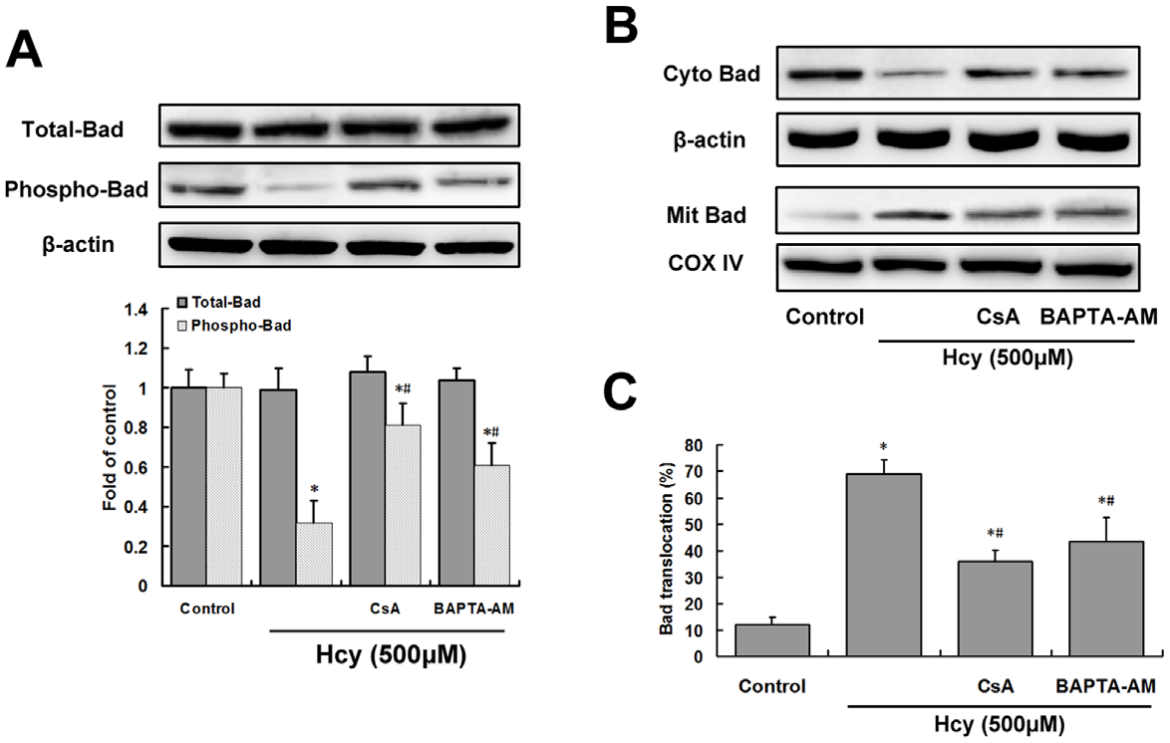
Calcineurin activation contributes to Hcy-induced dephosphorylation and mitochondrial translocation of Bad. Cells were pretreated with 1 µM CsA or 10 µM BAPTA-AM for 30 min before treatment with 500 µM Hcy (*Hcy*) for 24 h. A, Total Bad and phospho-Bad levels were assessed by western blotting and densitometric analysis. B, Western blot analysis of Bad in mitochondrial (Mit) and cytoplasmic (Cyto) fractions from hippocampal neurons. C, Percentage of total Bad (cytoplasmic +mitochondrial fractions) localized to the mitochondrial fraction was determined by densitometric analysis of the data from B. Results are expressed as the mean ± S.E.M. of three experiments. *P<0.05 vs. control. ^#^ *P*<0.05 vs. Hcy treatment group.

### Calcineurin Phosphatase Assay

Following treatments, cells were collected by centrifugation and total proteins were extracted as detailed above. Calcineurin activity was detected using the Calcineurin Phosphatase Assay Kit according to the manufacturer’s instructions (Enzo Life Sciences Inc., Farmingdale, NY). Calcineurin phosphatase activity was expressed as percentage of control.

### Recombinant Lentiviruses

Recombinant lentiviruses expressing the coding regions of green fluorescent protein (GFP) and rat 14-3-3ε were generated. The 14-3-3ε cDNA was cloned into the modified pLenti6/V5-DEST vector (Invitrogen). Recombinant lentiviruses were prepared by transfecting the pLenti6/V5-DEST plasmid along with packaging plasmids (Invitrogen) into HEK-293FT cells. Lentiviruses were concentrated by centrifugation of the lentiviral supernatant at 20,000 *g* at 4°C for 2 h and the virus pellets were resuspended in culture medium at a multiplicity of infection (MOI) of 10. Transduction of hippocampal neurons was conducted by the addition of 100 µl of lentiviral supernatants. After 24 h, the medium was replaced with fresh growth medium and antibiotic selection (2 µg/ml blasticidin) was initiated following an additional 2-day recovery. Lentiviral vector expressing GFP was used as control.

**Figure 5:**
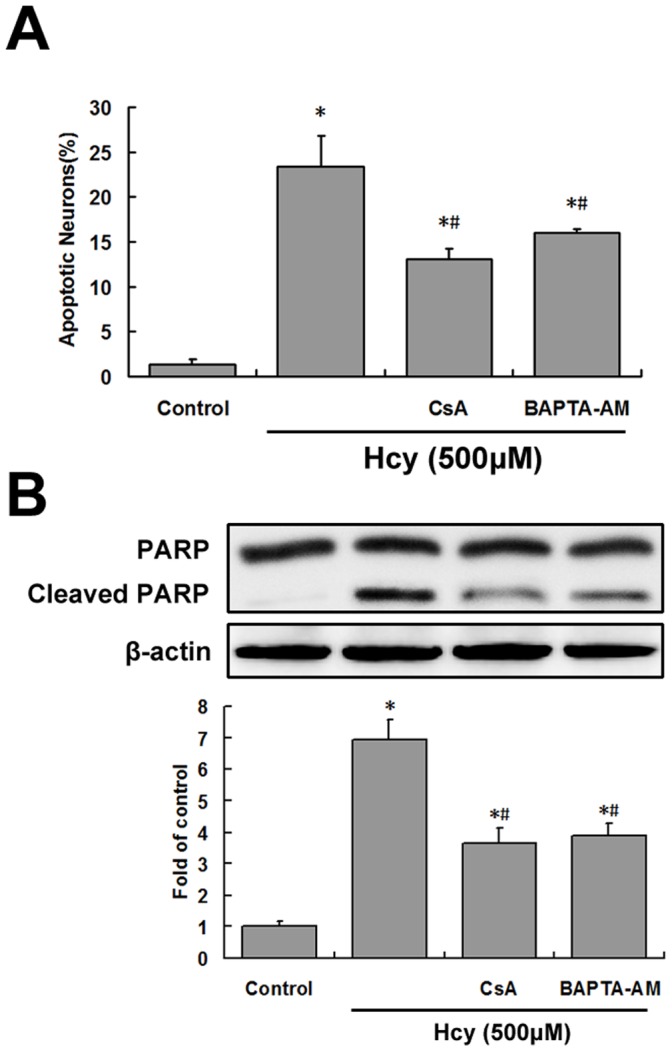
Inhibition of Hcy-induced apoptosis by a calcineurin inhibitor or intracellular Ca^2+^ chelator. Cells were pretreated with 1 µM CsA or 10 µM BAPTA-AM for 30 min before treatment with 500 µM Hcy (*Hcy*) for 24 h. A, The level of apoptosis was determined by counting the number of neurons with apoptotic nuclei. Four randomly selected fields of Hoechst-stained cells were counted and the average percentage of apoptotic cells per total number of cells was determined. B, Cleaved PARP was studied by western blotting. Results are expressed as the mean ± S.E.M. of three experiments. *P<0.05 vs. control. ^#^ *P*<0.05 vs. Hcy treatment group.

### Statistics

Each experiment was repeated a minimum of three times and results were expressed as the mean ± S.E.M. One-way ANOVA with the post-hoc Fisher’s test for multiple comparisons was used to examine the statistical differences between treatments. Differences were considered significant at *P<*0.05.

## Results

### Hcy Suppresses 14-3-3ε Expression

Exposure of primary hippocampal neurons to Hcy resulted in apoptosis in a concentration-dependent manner (Fig. 1A, B). Cleavage, and thus inactivation, of PARP by cysteine proteases of the caspase family is believed to play an important role in promoting apoptosis and preventing necrosis [23]. PARP cleavage was thus assessed by western blot to determine whether this key regulator of apoptosis is involved in Hcy-induced apoptosis. Cleavage of PARP to the smaller 85-kDa fragment, a process that is facilitated by caspase-3 activation, was present in neurons treated with Hcy and showed a dose-dependent response (*P<*0.05) while the amount of full-length PARP had slight changes; untreated controls did not show PARP cleavage (Fig. 1C). In addition, the levels of 14-3-3ε mRNA and protein, as measured by real-time quantitative PCR and western blotting, respectively, were significantly reduced by 250 µM and 500 µM Hcy (*P<*0.05; Fig. 1D).

**Figure 6:**
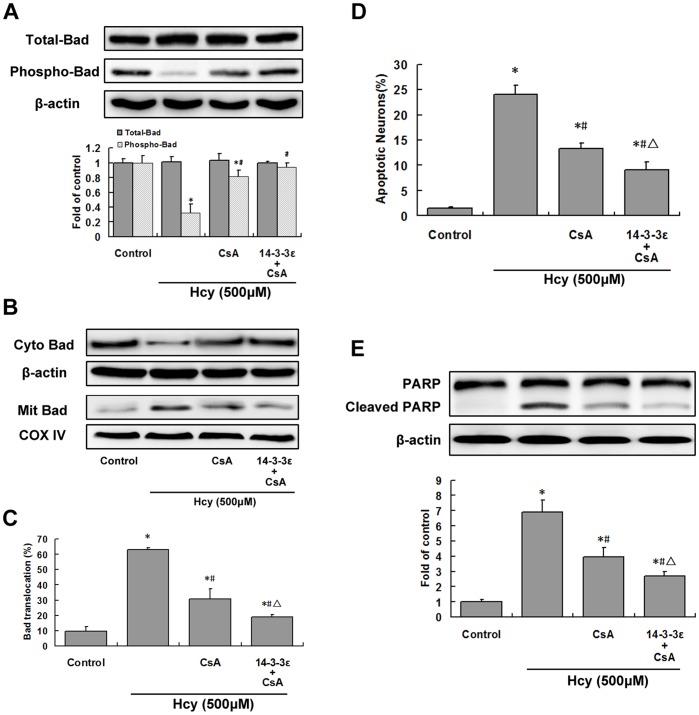
Calcineurin activation and 14-3-3ε suppression together contribute to Hcy-induced apoptosis. Hippocampal neurons transduced with lentiviral 14-3-3ε for 48 h were then pretreated with 1 µM CsA for 30 min before treatment with 500 µM Hcy (*Hcy*) for 24 h. A, Total Bad and phospho-Bad levels were assessed by western blotting and densitometric analysis. B, Western blot analysis of Bad in mitochondrial (Mit) and cytoplasmic (Cyto) fractions from hippocampal neurons. C, Percentage of total Bad (cytoplasmic+mitochondrial fractions) localized in the mitochondrial fraction as determined by densitometric analysis of the data from B. D, The level of apoptosis was determined by counting the number of neurons with apoptotic nuclei. Four randomly selected fields of Hoechst-stained cells were counted and the average percentage of apoptotic cells per total number of cells was determined. E, Cleaved PARP was measured using western blotting. *P<0.05 vs. control. ^#^ *P*<0.05 vs. Hcy treatment group. ^Δ^ *P*<0.05 vs. CsA group.

### Lentiviral 14-3-3ε Transduction Failed to Prevent Most of the Apoptosis

To examine whether overexpression of 14-3-3ε could prevent the apoptosis induced by Hcy, 14-3-3ε lentiviruses were generated. Hippocampal neurons transfected with these lentiviruses showed significant up-regulation of 14-3-3ε expression (*P<*0.05; Fig. 2A). 14-3-3ε overexpression slightly, but significantly, decreased apoptosis induced by Hcy exposure in comparison to that of the control group (*P<*0.05; Fig. 2B). The inhibitory effect of 14-3-3ε overexpression on apoptosis was supported by reduced levels of the PARP cleavage product (*P<*0.05; Fig. 2C). As the principal method used by 14-3-3ε to block apoptosis is by sequestration of phosphorylated Bad, total and phosphorylated Bad levels were studied; there were, however, no marked variations in total Bad levels, while phosphorylated Bad slightly, but not significantly, increased after 14-3-3ε lentivirus transfection (*P>*0.05; Fig. 2D).

### Hcy Activates Calcineurin

Dephosphorylation of Bad has been proven to promote apoptosis [20]. Since up-regulation of 14-3-3ε did not reverse the reduction in phosphorylated Bad evoked by Hcy, there must be other mechanisms that mediate dephosphorylation of Bad in hippocampal neurons exposed to Hcy. Therefore we measured the activity of calcineurin, a Ca^2+^/calmodulin serine/threonine phosphatase that dephosphorylates Bad in glutamate-induced apoptosis [20]. As shown in Fig. 3A and B, Hcy caused a concentration and time-dependent increase in cellular calcineurin activity. The increased activity of calcineurin was blocked by the calcineurin inhibitor CsA (Fig. 3B). Calcineurin activation was related to an increase in cytosolic free Ca^2+^, as BAPTA-AM, an intracellular Ca^2+^ chelator, also blocked calcineurin activation (*P<*0.05; Fig. 3B). We also directly measured the calcineurin protein levels by Western Blot with antibody that detects calcineurin A subunit. As shown in Fig. 3C, no differences in calcineurin A protein content were identified between neurons treated with or without Hcy (500 µm) and that pretreated with CsA or BAPTA-AM.

### Activated Calcineurin Contributes to Dephosphorylation and Translocation of Bad

In cultures treated with Hcy, the levels of phosphorylated Bad were found to be significantly reduced (*P*<0.05) compared to those in the control group, while the levels of total Bad showed no significant changes (Fig. 4A). Pretreatment with either CsA or BAPTA-AM had no effect on total Bad levels but significantly blocked the reduction in phosphorylated Bad evoked by Hcy alone (*P*<0.05; Fig. 4A). After treatment with Hcy (500 µM), Bad underwent redistribution from the cytosol to the mitochondria (Fig. 4B, C). However, in the presence of calcineurin inhibitors, Hcy-induced Bad translocation was significantly decreased (*P*<0.05; Fig. 4B, C).

### Activated Calcineurin Contributes to Hcy-induced Apoptosis

To further confirm the link between calcineurin activation and neuronal apoptosis, the apoptotic level in Hoechst-stained cultures was quantified. Apoptosis was blocked by CsA or BAPTA-AM pretreatment (*P*<0.05; Fig. 5A), and pretreatment with the calcineurin inhibitors significantly decreased the levels of cleaved PARP (*P*<0.05; Fig. 5B).

### Calcineurin Activation and 14-3-3ε Suppression in Hcy-induced Apoptosis

To determine whether calcineurin activation in conjunction with 14-3-3ε inhibition contribute to apoptosis induced by Hcy, hippocampal neurons transduced with lentiviral 14-3-3ε were treated with the calcineurin inhibitor, CsA. Lentiviral 14-3-3ε in combination with CsA significantly increased phosphorylated Bad (*P*<0.05; Fig. 6A), and decreased mitochondrial Bad translocation to a greater extent than calcineurin inhibitor treatment alone (*P*<0.05; Fig. 6B, C). The levels of apoptosis, assessed by Hoechst staining, and cleaved PARP, determined by western blotting, also indicated that the combination of lentiviral 14-3-3ε with CsA showed a synergistic effect (*P*<0.05; Fig. 6D, E).

## Discussion

To investigate the mechanism of Hcy-induced apoptosis in hippocampal neurons, alterations in 14-3-3ε expression, calcineurin activity and Bad dephosphorylation were examined. In addition, the roles of lentiviral 14-3-3ε and calcineurin inhibitors in suppressing apoptosis were evaluated. Two major findings of this study are that Hcy induces apoptosis in rat hippocampal neurons via calcineurin-mediated dephosphorylation of Bad, and that 14-3-3ε overexpression in combination with the calcineurin inhibitor, CsA, synergistically inhibits Hcy-induced apoptosis.

Reduction of 14-3-3ε expression was detected recently by proteomic analysis in a neonatal rat hypoxia/ischemia model [24], but, to the best of our knowledge, this has not been reported in hyperhomocysteinemia. Recent evidence suggests that 14-3-3ε can rescue brain tissue and N2A cells from ischemia-induced damage and apoptosis by promoting the sequestration of phosphorylated Bad [25]. Increased 14-3-3 expression also shows a neuroprotective effect in Parkinson’s disease models [15]. In the present study, we found that high concentrations of Hcy (250 and 500 µM) could suppress the expression of 14-3-3ε in hippocampal neurons, while lentiviral 14-3-3ε transduction could only slightly inhibit apoptosis, suggesting that there is another mechanism involved in Hcy-induced apoptosis.

Previous studies suggest that Hcy enhances NMDA receptor function and mobilizes intracellular calcium stores [9]. The current results link the increase in Ca^2+^ to activation of calcineurin, which in turn dephosphorylates Bad to stimulate its translocation to mitochondria. The subcellular location of Bad is controlled by its phosphorylation status. It exists in a phosphorylated state in the cytoplasm, bound to 14-3-3, and in a non-phosphorylated state at the mitochondria surface, where it appears to neutralize the anti-apoptotic effects of Bcl-2 and Bcl-xL [26], [27], [28]. As downstream events after Bad dimerization with Bcl-xL, Bax translocation to mitochondria and cytochrome *c* translocation to the cytoplasm have been shown to occur in hyperhomocysteinemia in the hippocampus [18].

Taken together, the suppression of 14-3-3ε and the activation of calcineurin may cooperate in Hcy-induced apoptosis. The dephosphorylation of Bad induced by activated calcineurin promotes Bad translocation to mitochondria which, due to a lack of Bad sequestration, may be reinforced by the reduction of 14-3-3ε. The results of this study showed that lentiviral 14-3-3ε in conjunction with calcineurin inhibitors increased phosphorylated Bad and decreased Bad translocation to a greater extent than in the calcineurin inhibitor-alone groups. Apoptosis was also more robustly inhibited in the combined group than in the separate groups.

Hcy may have multiple effects on neuronal cells, depending on cell type, Hcy exposure time and concentration, and the microenvironment [10]. For example, Hcy may exhibit either neuroprotective activity or neurotoxic attributes in cultured rat cerebrocortical neurons depending on glycine concentrations [9]. In the presence of glutamate or copper, Hcy acts as an activator and potentiates their neurotoxicity [8], [29]. The Ca^2+^ influx and calcineurin activation shown in the present study are not involved in all examples of Hcy-induced neurotoxicity, such as acute Hcy toxicity (30 min, 25 mM Hcy) [30], [31]. Therefore, Bad-mediated mitochondrial apoptosis may be one of a number of mechanisms through which Hcy induces neurotoxicity.

In summary, the present study confirms that Hcy suppresses 14-3-3ε expression and activates calcineurin in rat hippocampal neurons. Calcineurin-mediated Bad dephosphorylation is an upstream event that leads to Bad translocation and apoptosis that is synergistically suppressed by 14-3-3ε.
